# Emotional and non-emotional pathways to impulsive behavior and addiction

**DOI:** 10.3389/fnhum.2013.00043

**Published:** 2013-02-21

**Authors:** Ana Torres, Andrés Catena, Alberto Megías, Antonio Maldonado, Antonio Cándido, Antonio Verdejo-García, José C. Perales

**Affiliations:** ^1^Learning, Emotion, and Decision Research Group, Mind, Brain and Behavior Research Center/Centro de Investigación Mente, Cerebro y Comportamiento (CIMCYC), Universidad de GranadaGranada, Spain; ^2^Departamento de Personalidad, Evaluación y Tratamiento Psicológico, Universidad de GranadaGranada, Spain; ^3^School of Psychology, Psychiatry and Psychological Medicine, Monash UniversityMelbourne, VIC, Australia

**Keywords:** impulsivity, emotion, addiction, decision-making, delay discounting, Go/No-go, UPPS-P, N2 ERP

## Abstract

Impulsivity is tightly linked to addiction. However, there are several pathways by means of which impulsive individuals are more prone to become addicts, or to suffer an addiction more intensely and for a longer period. One of those pathways involves an inadequate appraisal or regulation of positive and negative emotions, leading to lack of control over hazardous behaviors, and inappropriate decisions. In the present work, we assessed cocaine-dependent individuals (CDI; *n* = 20), pathological gamblers (PG; *n* = 21), and healthy controls (HC; *n* = 23) in trait impulsivity measures (UPPS-P model's dimensions), and decision-making tasks (Go/No-go; delay-discounting task). During the Go/No-go task, electroencephalographic (EEG) activity was recorded, and Go/No-go stimuli-evoked potentials (ERP) were extracted. Theory-driven ERP analyses focused on the No-go > Go difference in the N2 ERP. Our results show that negative urgency is one of the several psychological features that distinguish addicts from HC. Nevertheless, among the dimensions of trait impulsivity, negative urgency is unique at independently covarying with gambling over-pathologization in the PG sample. Cocaine-dependent individuals performed more poorly than gamblers in the Go/No-go task, and showed abnormal Go/No-go stimuli-evoked potentials. The difference between the No-go stimulus-evoked N2, and the Go one was attenuated by severity and intensity of chronic cocaine use. Emotional dimensions of impulsivity, however, did not influence Go/No-go performance.

## Introduction

Substance and behavioral addictions are characterized by loss of control over drives or habits, and by persistent preference for immediate rewards at the expense of relative net loss (Everitt and Robbins, 2000). Behavioral neuroscience has modeled these deficits by using motor inhibition (or impulsive action) and delay discounting (or impulsive choice) decision-making tasks (Winstanley et al., 2006; Verdejo-García et al., 2008). However, there are at least two mechanisms potentially accounting for impulsive actions and/or choices in addicts: one refers to pre-existing individual differences within impulsive personality traits, and the other to the impact of the degree of exposure to drug dosages or gambling episodes.

With regard to the first mechanism, there are multiple facets of trait impulsivity that may impact impulsive action and choice, and thus addiction vulnerability. The UPPS-P model of impulsivity has recently emerged as a successful factorial account of impulsive personality (Whiteside and Lynam, 2001; Cyders and Smith, 2008; Cándido et al., 2012). In this model, impulsivity is hypothesized to be composed of five separable dimensions. *Negative urgency* refers to the tendency to make poor decisions under conditions of negative affect; *positive urgency* refers to the tendency to make poor decisions under conditions of positive affect; *lack of premeditation* is the tendency not to think of the consequences of an action before engaging in it; *lack of perseverance* refers to the inability to stay focused on long, boring or difficult tasks; and *sensation seeking* represents the willingness to participate in exciting, new, and/or potentially dangerous activities. Most importantly, impulsivity dimensions in this model have been proved to correlate with future (prospective) aspects of addictive behavior, in a meaningful manner (see, for example, Cyders et al., 2007, 2009; Cyders and Smith, 2008).

With regard to the second mechanism, prolonged stimulants or gambling exposure can gradually boost impulsive traits and deter cognitive performance in response inhibition and delay discounting tests. In terms of behavioral performance, cocaine users seem disproportionately impaired in response inhibition tests (Verdejo-García et al., 2007b), whereas pathological gamblers (PG) display consistent preference for immediate rewards, in absence of significant influence of response inhibition deficits (Kertzman et al., 2011).

In other words, addiction, trait impulsivity, and performance in delay discounting and response inhibition tasks compound quite a complex, multifaceted triad, in which disentangling causes from effects is not always easy. The most straightforward way to do so is by means of prospective studies, in which personality and neuropsychological scores are used to predict the future emergence of addictive disorders. Cross-sectional studies, comparing addicts vs. non-addicts, are more limited in this regard. Nevertheless, in groups of addicts, it is possible to check for the existence of functional relations between the degree of exposure to the potentially neurologically damaging factor (e.g., the amount of drug consumed during the course of addiction) and the extent of personality and neuropsychological anomalies (including impulsive choice and impulsive action in lab tasks, and their neurophysiological correlates). Relationships of this type are strongly indicative of neurotoxic and/or neuroadaptive effects (see Albein-Urios et al., 2012a, for a more detailed presentation of this argument).

In the present work, we compared a group of PG against a group of cocaine dependent individuals (CDI), and a group of matched healthy controls (HC), in trait impulsivity dimensions (UPPS-P), impulsive choice (delay discounting), and impulsive action (motor inhibition Go/No-go task). Our first aim is to help to clarify the current state of affairs by carefully estimating the degree of exposure to cocaine/gambling activities, and checking for relationships, not only between the clinical category and personality/impulsive choice/impulsive action anomalies, but also between those anomalies and exposure estimates, within the clinical categories.

Complementarily, we have solid reasons to believe that some light can be shed on this complex pattern of interrelations by drawing two paths in it: one based on *emotion* appraisal and processing, and a second, *non-emotional* one, mostly related to other (“cool”) components of executive functioning (Metcalfe and Mischel, 1999). On this regard, our first working assumption is the centrality of emotion in the characterization of different types of impulsivity. The segregation between emotional and non-emotional components of impulsivity has indeed a long history (see Barratt, 1993; Evenden, 1999; Perales et al., 2009), and is also implicit in the UPPS-P model (Whiteside and Lynam, 2001; Cyders et al., 2007; Cyders and Smith, 2008). Negative urgency, positive urgency, and sensation seeking, are by definition strongly loaded by emotional factors (Joseph et al., 2009). Among them, positive and negative urgency depend on inadequate appraisal of (and response to) emotions preceding decisions, whereas sensation seeking is more related to the anticipation of reward and the lack of apprehension against the risks involved in its attainment. The other two dimensions—lack of perseverance and lack of premeditation—are less dependent on emotion. In that sense, the UPPS-P model emerges as a tool to identify the importance of emotional vs. non-emotional ingredients of impulsivity in all kinds of hazardous, risky or pathological behavior.

More generally speaking, emotion is linked to impulsive behavior in at least two ways: via *behavior consequences*, and via *behavior antecedents*. In support of the first link, there is evidence of a psychological link between reward/punishment and emotion, so that the neural substrates for them are partially overlapping (Kringelbach, 2005; Murray, 2008; Quartz, 2009). Some individuals can be specially (in)sensitive to the affective value of present or delayed reward and punishment, so that, for example, punishment-sensitive people can be less prone to take risks, or reward-sensitive people can be more adventurous and sensation seeking (Dawe and Loxton, 2004; Franken and Muris, 2006; Reynolds, 2006; Torres et al., submitted). In the second case, emotions, acting as context for decisions, can turn such decisions into impulsive ones. Recent studies (see, for example, Albein-Urios et al., 2012b; Verdejo-García et al., 2013) report strong evidence on the involvement of inadequate appraisal of emotions (particularly negative ones) in hazardous behavior.

Our second working assumption is that the two neurobehavioral mechanisms theoretically linking brain function to rash action and addiction (delay discounting sensitivity and motor inhibition) are also differentially related to emotions. Separate pieces of previous evidence seem to support this idea. For example, Metcalfe and Mischel (1999) interpret findings from reward delay studies in terms of a hot/cool systems theory. The results reviewed support the idea that increased “cool” system activation increases the ability to delay gratification, whereas increased “hot” system activation decreases it. Importantly, emotions generated incidentally at the time of decision, but not directly related to the expected, to-be-delayed reward, as well as emotions consubstantial to the object of decision, can significantly influence choice (Schmeichel and Inzlicht, 2013).

On the other hand, core motor inhibition mechanisms seem to be mostly non-emotional, although this statement is more controversial. A broad exploration of the cognitive processes and brain areas involved in Go/No-go and other inhibition tasks can be found in Rubia et al. (2001). The areas identified comprise parts of the frontal, prefrontal, and parietal cortices, mostly—although not exclusively—coincident with the “cool” executive system (although see also Goldstein et al., 2007). According to Horn et al. (2003), differential Go/No-go stimulus-evoked early processing is mostly non-emotional, and reflects the core inhibitory component of the task. Conversely, more delayed processing recruits limbic and paralimbic structures (posterior orbitofrontal cortex, the temporal poles, and the posterior cingulate) which are likely to implement the emotional and motivational aspects of the task. Given this somewhat mixed pattern of results, providing correlations between different aspects of Go/No-go performance and emotional and non-emotional dimensions of impulsivity is theoretically valuable.

To sum up, with the present work we intend to add evidence on the two abovementioned assumptions and, complementarily, to make them converge to test the differential involvement of the emotional and non-emotional pathways in the two addictive disorders under scrutiny. With that intention in mind, we interviewed participants in their present and past drug-using and gambling habits, and we used the UPPS-P model to evaluate impulsive personality in the three groups, the Now-or-later test (Kirby et al., 1999) for delay discounting/impulsive choice; and the Go/No-go task (Verdejo-García et al., 2007b) for motor inhibition/impulsive action.

In addition, during the Go/No-go task, electroencephalographic (EEG) activity was recorded, and Go/No-go stimuli-evoked potentials (ERP) were extracted. Theory-driven ERP analyses focused on the N2 component, as observed in frontocentral electrodes (Jodo and Kayama, 1992; Mathalon et al., 2003; Miltner et al., 2003; Beste et al., 2011; Smith, 2011; Gajewski and Falkenstein, 2013) as the potentially best candidate to reflect inhibition-related cognitive activity during the Go/No-go task (Mathalon et al., 2003; see Folstein and Van Petten, 2007, for a review). Moreover, there are numerous reports of the relationship between N2 magnitude and addictive behavior, and, specifically, a reduction of the No-go > Go difference and the underlying source activations in samples of clinically addicted patients (Sokhadze et al., 2008; Dong et al., 2010; Luijten et al., 2011; Pandey et al., 2012). Complementarily, ERP measures are frequently more sensitive than behavioral ones, and can provide convergent evidence of the effect of key manipulations, especially when the behavioral effects of such manipulations are subtle (see, for example, Karayanidis et al., 2000; Hajcak et al., 2005).

For analysis and presentation purposes, our aim is divided into three specific research targets: (1) To quantify the influence of emotional and non-emotional dimensions of impulsivity, as measured by the UPPS-P model, on cocaine-use and gambling. Impulsivity dimensions are used, to *postdict* belongingness to clinical categories, but also to check for correlations with gambling episodes/cocaine dosage exposure estimates. (2) To check for the existence of differences between the clinical and non-clinical groups, as well as exposure-mediated effects on performance in Go/No-go and delay-discounting tasks (and on evoked EEG activity during the Go/No-go task). Finally (3), and in order to add evidence on the linkage between impulsive choice/action and emotional/non-emotional dimensions of impulsivity, we measured the differential impact of such dimensions on the two decision-making tasks, without taking addiction into account. The consequences of our results on the triadic mapping between impulsivity, decision-making, and addiction, and the role of emotions (particularly negative ones) in that mapping, will be discussed.

## Materials and methods

### Participants

Cocaine-dependent individuals (CDI; *n* = 20) were recruited from the Proyecto Hombre rehabilitation centers in Granada and Málaga (Spain) between January 2011 and July 2012. Pathological gamblers (PG; *n* = 21) were recruited from AGRAJER (Granadian Association of Gamblers in Rehabilitation, Granada, Spain) between October 2010 and July 2012. Most controls (*n* = 23) were recruited among non-drug using, non-gambling partners, and acquaintances (with no familiar linkage) of individuals in the clinical groups. The rest of them were recruited by incidental sampling, in such a way that their sociodemographic characteristics were not far from the clinical groups.

The inclusion criteria were (i) meeting DSM-IV criteria for cocaine dependence (CDI group) or pathological gambling (PG group)—as assessed by the Structured Clinical Interview for DSM-IV Disorders—Clinician Version (SCID; First et al., 1997); (ii) having a minimum abstinence interval of 15 days for all substances of abuse except nicotine, as determined by weekly urine toxicological tests (CDI) or cross validated therapist- and self-reports (PG). Exclusion criteria were: (i) the presence of any other Axis I or Axis II comorbid disorders with the exception of nicotine dependence; (ii) the presence of history of head injury or any diseases affecting the central nervous system.

The participants volunteering in this study were the same as those in Torres et al. (submitted). The specific socio-demographic characterization of the three groups is reported in Torres et al.'s abovementioned study. For the HC, PG, and CDI groups, respectively, sample sizes were 23, 21, and 20; proportions of females 0.09, 0.10, and 0.00; mean (SD) age 30.13 (8.64), 31.43 (5.92), and 34.75 (6.51); mean (SD) education years 14.55 (3.16), 13.90 (4.66), and 15.05 (4.21); and mean (SD) IQ 106.25 (10.22), 101.10 (9.07), and 105.35 (9.09). In all cases, differences between groups were not significant (min *p* = 0.11).

The study counted with explicit permission from the University of Granada's ethics committee. Prior to psychological and neuropsychological assessment, all participants were informed about the objectives and characteristics of the study, and signed an informed consent form. All of them were compensated with 36 € for their participation, independently of performance.

Upon consent, all participants were questioned about their age and number of education years, and were assessed using the Kaufman Brief Intelligence Test (K-BIT), the Interview for Research on Addictive Behavior (IRAB), the UPPS-P impulsivity scale, the Now-or-later task, and the Go/No-go task.

### Psychometric instruments

#### UPPS-P impulsive behavior scale (Verdejo-García et al., 2010)

In order to assess impulsivity, we used the Spanish version of the original 59-item questionnaire (Verdejo-García et al., 2010). This scale allows for a multidimensional assessment of impulsivity: positive, urgency, negative urgency, (lack of) premeditation, (lack of) perseverance, and sensation seeking.

#### Interview for research on addictive behaviors (IRAB, Verdejo-García et al., 2005)

As noted in the introduction, a key factor in the present study is the degree of exposure to cocaine and gambling activities (in the CDI and PG groups, respectively). Most psychometric tools developed for clinical purposes do not measure exposure in an isolated manner (disregarding craving intensity, perception of lack of control over the addictive behavior, social and family problems, financial problems, and other symptoms and consequences of addiction).

All of those side factors are irrelevant to the current study. Actually, they would likely blur drug/gambling exposure effects. Hence, information about lifetime amount and duration of use of the different drugs was collected using the IRAB (Verdejo-García et al., 2005). The IRAB is inspired by applied and experimental behavior analysis, and was not developed to estimate the clinical significance of addiction, but to quantify the most important parameters of drug use behaviors (frequency, duration, amount), independently of the clinical status of the participant and the accompanying symptomatology. All the participants in the three groups went through the full IRAB interview. Here, however, we will consider only two composite measures yielded by the interview: (1) monthly amount of each drug consumed, in grams/month, and (2) severity, or estimated lifetime amount of drug consumed. In order to avoid extremely skewed distributions, monthly amount and severity were translated into within-design rank scores for all analyses. A more detailed display of IRAB results for the three groups can be found in Torres et al. (submitted, Appendix 1).

The IRAB has not been yet developed for gambling activities, so, in order to have equivalent measures for gambling and cocaine use, gamblers were questioned about the amount of money they used to gamble per month (in euros), and the lifetime duration of regular gambling (in months), across the whole course of their addiction. That is, the same questions used in the IRAB for registering drug use, were adapted to register the two key gambling parameters. Following the same procedure used with cocaine use parameters, severity of gambling was computed as the product of such factors, and then translated into within-design rank scores.

#### The Kaufman Brief Intelligence Test (K-BIT, Kaufman and Kaufman, 1990)

The K-BIT has been standardized and utilized widely, in both clinical and research settings, to assess cognitive abilities. It comprises measures of verbal and non-verbal intelligence and takes 10–30 min to administer. For control matching purposes, we will use only the compound IQ total score.

### Decision-making tasks

#### Go/No-go

A computer-based implementation of the Go/No-go task was used (Verdejo-García et al., 2007b). The task consisted of 200 trials. In the first 100 trials (pre-switch), participants were asked to press any key as quickly as they could whenever the Go stimulus (a letter) was presented, and to withhold the response when the No-go stimulus (a different letter) was presented. The assignation of stimuli to the Go and No-go conditions was counterbalanced across subjects. In the second 100 trials of the task (post-switch), participants were asked to switch the assignation of the response from the Go to the No-go stimulus; in other words, they were asked to respond to the previously No-go stimulus and not to respond to the previously go stimulus. The proportion of Go vs. No-go trials on both phases (pre- and post-switch) was 80/20. The inter-trial interval (ISI) was set at 100 ms, and each stimulus was presented during 1000 ms. Auditory feedback (one of two distinctive sounds) was provided after each response to indicate whether that response had been right or wrong. If participants did not respond in the 1000 ms response window, the two same sounds were used as positive and negative feedback for not responding. That is, if no response was given, the same sounds indicated whether the absence of response had been right (No-go trials) or wrong (Go trials). Participants were instructed to respond as quickly as they could; however, in order to enforce time pressure, all responses given more than 400 ms after the stimulus were accompanied by the message “late,” displayed on the center of the screen.

This version of the task was longer, but virtually identical to the one used by Verdejo-García et al. (2010). In previous implementations and pilot studies with the task, we have observed that false alarms (commission errors) and misses (omission errors) tend to become less frequent as the task progresses, which tends to generate performance floor and ceiling effects. The switch between phases was included to prevent such effects and boost the task's sensitivity to manipulations. Similarly, time pressure on responses (400 ms) prevents floor effects in false alarm rates' analyses, and tends to increase the magnitude of the No-go > Go N2 difference (Sokhadze et al., 2008).

#### Now-or-later (Kirby et al., 1999)

This paper-and-pencil task presents participants with 27 hypothetical two-option choices between an immediate small reward, and a delayed larger one (e.g., would you prefer 55 € now, or 110 € in 15 days?). The 27 items are pairs of imaginary monetary rewards varying in amount. Although responses allow for the calculation of the discount parameter (*k*), namely, the rate at which reward looses subjective utility across time, the calculation of such a parameter requires certain assumptions about the best-fitting time-utility function. Among the several theory-free possible dependent measures, we selected the simplest one, the total number of decisions favoring the immediate reward, as the main measure of reward-delay sensitivity.

### ERP extraction and analysis

EEG activity was recorded exclusively during the Go/No-go task. EEGs were recorded from 62 scalp locations using tin electrodes arranged according to the extended 10–20 system mounted on an elastic cap (Brain Products, Inc.), and referenced online to FCz. Vertical and horizontal eye activity were recorded from one monopolar electrode placed below the left eye, and one monopolar electrode located in a straight line at the outer canthi of the right eye. Two scalp electrodes were attached to mastoids. All electrode impedances during recording were below 5 kΩ. EEG and EOG were sampled at 1000 Hz and amplified using a 0.016–1000 Hz band-pass filter. Subsequently, all EEG recordings were downsampled to 250 Hz, band-pass filtered using a 0.1–25 Hz 12 db/octave, re-referenced offline to average activity of the mastoids electrodes, and FCz activity was recovered. Offline signal preprocessing was done using EEGLAB software (Delorme and Makeig, 2004; freely available at http://sccn.ucsd.edu/eeglab).

EEG recordings were segmented from −200 to +300 ms, time-locked to the Go/No-go stimulus onset. Epochs were corrected for ocular artifacts by first computing the SOBI ICA decomposition (Belouchrani et al., 1993, 1997; Cardoso and Souloumiac, 1996, see also Tang et al., 2004) as identified by the ADJUST algorithm (Mognon et al., 2011). Other artifacts were subsequently removed using an automatic rejection procedure: segments were excluded for the remaining analyses when amplitudes were outside the ±100 μV range. Afterwards, segments were categorized as belonging to the Go or the No-go conditions. After the artifact correction procedure a minimum of 12 trials for the No-go and 57 for Go segments were retained for further processing.

The N2 amplitude was computed as the peak-to-peak difference between the most positive peak in the 160–220 ms time window and most negative peak in the 240–300 ms time window (see Nieuwenhuis et al., 2003; Smith, 2011, for a similar procedure). Given that the No-go > Go N2 difference is especially neat over frontocentral electrodes and independent of the reference (Jodo and Kayama, 1992; Mathalon et al., 2003; Miltner et al., 2003; Yeung and Nieuwenhuis, 2009; Smith, 2011), we used Fz, FCz, and Cz for testing between-group differences.

### Source location

Standardized Low-Resolution Electromagnetic Tomography (sLORETA) was used for estimating the 3-D cortical distribution of current density underlying scalp activity. sLORETA, computations were done using the MNI152 template, with the 3D space solution restricted to cortical gray matter, according to the probabilistic Talairach atlas (Talairach and Tournoux, 1988). The cortical gray matter is partitioned in 6239 voxels at 5 mm spatial resolution. Brodmann anatomical labels are reported using MNI space. Standardized sLORETA current source densities with no regularization method were obtained from 61 channels (after recovering FCz) for each participant in each condition and for each time point in each feedback condition.

Brain localization analysis was carried out according to the following steps: first, a single measure of the activation of each voxel for the N2 interval (240–300 ms) was computed, by averaging voxel activations across the whole interval, and correcting by the average activity at the 160–220 interval. Second, we computed the correlation (across participants) between that averaged current density and the magnitude of the No-go > Go N2 difference, for each voxel and each feedback condition. And third, those areas in which at least 10 voxels were found to significantly correlate with the No-go > Go N2 difference score were identified. For a more detailed description of this source location rationale see Catena et al. (2012). For a virtually identical combination of peak-to-peak N2 amplitude computation, and time-window averaging for current source density estimation, see Nieuwenhuis et al. (2003).

## Results

### Relationships between impulsivity dimensions and addictive behavior

#### Impulsivity dimensions and belongingness to clinical groups

Forward stepwise binomial regression analyses were carried out to test whether impulsivity dimensions were indicative of belongingness to the clinical groups. In the first one, the five UPPS-P dimensions entered the analysis as independent factors, and the clinical category [addicts (PG, CDI) vs. non-addicts (HC)] as dependent variable.

Stepwise regression is recommended over simultaneous regression for small samples. In the step 0 of this procedure the five impulsivity dimensions are included in a full regression model. In following steps, the variables are added one-by-one, in accordance with their predictive value. Only significant variables are introduced in the prediction equation. In our case, the process stopped at step 3, after including negative urgency, lack of premeditation and sensation seeking. The three-dimension binomial regression model correctly classified 82.8% of the cases. As displayed in Table 1, negative urgency, lack of premeditation, and sensation seeking were independently indicative of addiction. Both negative urgency and lack of premeditation were direct postdictors of addiction, whereas sensation seeking was a sign of non-addiction.

**Table 1:** **Forward binomial regression analysis (step 3) of clinical addiction [(CDI + PG) vs. HC] upon impulsivity dimensions**.

	***B***	***SE***	***Wald***	***p***
Negative urgency	1.084	0.334	10.517	0.001
Lack of premeditation	0.958	0.357	7.210	0.007
Sensation seeking	–0.746	0.340	4.801	0.028

B, standardized regression parameter; SE, standard error; Wald, contrast statistic; p, alfa error.

Secondly, a similar analysis was carried out to test whether impulsivity dimensions could distinguish between the two clinical categories (PG vs. CDI). Neither the full model nor any of the dimensions were indicative of the clinical category in this case (min *p* = 0.16).

#### Impulsivity dimensions and addiction severity

The model resulting from regressing gambling severity (in the PG group) over the five UPPS-P dimensions was significant (*R*^2^ = 0.25), but only negative urgency was included in it, [β = 0.50, *t*_(20)_ = 2.53, *p* = 0.02]. The other four dimensions were far from significance (min *p* = 0.22). In the case of cocaine dependent individuals, neither the full regression model nor any of the UPPS-P dimensions was significantly predictive of cocaine use severity (computed for the CDI only; min *p* = 0.27).

### Differences between clinical and non-clinical groups in decision-making tasks

#### Go/No-go task

***Behavioral results.*** A *hit rate* (*h*) was computed as the number of hits (responses to Go trials) divided by the sum of hits and misses (non-responses to Go trials), for each 25-trial block of the task. Similarly, a *false alarm rate* (*f*) was computed as the number of false alarms (responses to No-go trials) divided by the sum of false alarms and correct rejections (non-responses to No-go trials), for each 25-trial block of the task.

A group (HC, PG, CDI) × block (1–8) MANOVA on false alarm rates yielded a significant effect of group [Wilks' Λ = 0.63, *p* = 0.05]. Šidák-corrected *post-hoc* tests revealed a significant difference between PG and CDI in block 4 (*p* = 0.02), but not between PG and HC (*p* = 0.83), nor between HC and CDI (*p* = 0.25). Analogous group × block MANOVAs on hit rates and hit latencies did not yield any significant effect (min *p* = 0.46).

In order to test potential mediating effects of addiction severity we carried out MANCOVAs across blocks, with addiction severity as a continuous covariate, for mean false alarm rates, mean hit rates, and mean hit latencies, separately for PG and CDI. None of such analysis yielded any significant effect of severity or intensity (min *p* = 0.12).

**Figure 1:**
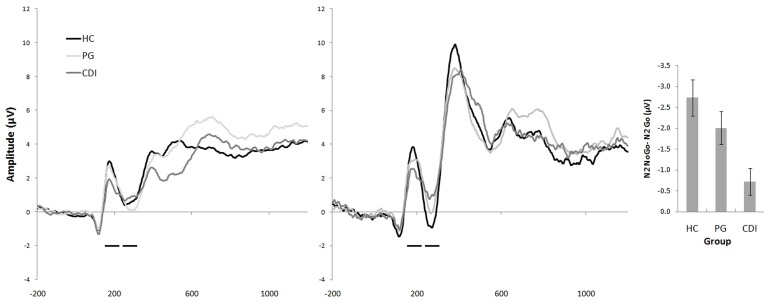
**ERP waveform across groups (HC: Healthy Controls, PG: Pathological gamblers, CDI: Cocaine-dependent individuals) at FCz, time-locked to the Go stimulus (left panel), and the No-go stimulus (middle panel).** The right panel displays the No-go > Go N2 difference (the difference in N2 amplitudes between Go and No-go stimuli) for the three groups. The marks indicate the time frames used for the computation of the No-go > Go N2 difference.

***N2.*** Figure 1 displays ERP waveforms, time-locked to the Go/No-go stimulus, separately for Go (left panel) and No-go stimuli (middle panel). The right panel represents the No-go > Go N2 difference for the three groups. No-go and Go N2 amplitudes were submitted to a 3 (group: HC, CDI, PG) × 2 (type of trial: No-go, go) × 3 (channel: FCz, Cz, Fz) repeated measures analysis of variance. Such an ANOVA yielded main effects of the type of trial, *F*_(1, 61)_ = 63.00, *MSE* = 5.01, *p* < 0.01, and its interaction with group, *F*_(2, 61)_ = 6.65, *p* < 0.01. No other main or interaction effects were significant. Analyses within the interaction yielded a group effect for the No-go condition, *F*_(2, 61)_ = 3.83, *p* < 0.03, but not for the Go one, *F*_(2, 61)_ = 0.05. Bonferroni-corrected *post-hoc* comparisons indicated that negative amplitudes were larger for HC than for CDI (*p* = 0.03). No differences were observed between HC and PG (*p* = 0.58), or between PG and CDI (*p* = 0.10). No-go vs. Go differences were significant for all the groups, *t*_(23)_ = 6.28, *p* < 0.01, *t*_(19)_ = 5.07, *p* < 0.01, and *t*_(19)_ = 2.24, *p* = 0.04, respectively, for HC, PG, and CDI groups.

Correlations between cocaine use severity and monthly use and the No-go > Go N2 difference were both significant (*r* = 0.40, *p* < 0.01, and *r* = 0.43, *p* < 0.01, for severity and intensity, respectively). Restricting the analysis to the CDI group, the differential N2 effect was correlated only with use intensity (*r* = 0.57, *p* < 0.01), but not with use severity (*p* = 0.45). Given that the No-go > Go N2 effect has a negative sign, the direct correlations indicate that severity and intensity attenuated the difference between the No-go stimulus-evoked N2, and the Go N2.

Gambling intensity and severity did not correlate with the differential N2 effect (independently of whether the analysis was performed for the whole sample, or only for the PG group; all *p* > 0.22).

**Figure 2:**
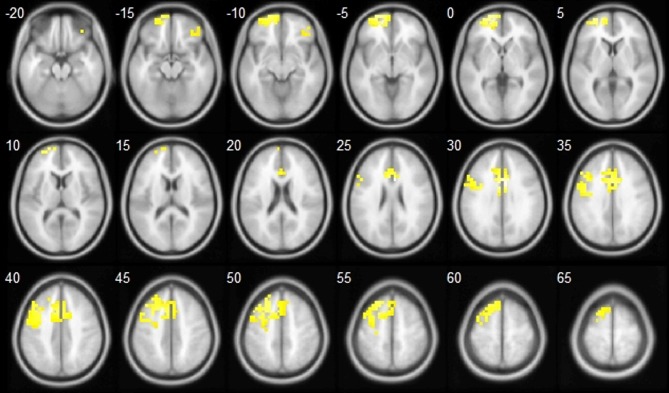
**Topographical localization of the cortical areas identified to significantly correlate with No-go > Go N2 difference scores in the whole sample of participants**.

***Source location.*** Using the bootstrapping approach to control for multiple comparisons, included in the sLoreta package, we observed several clusters of voxels that significantly correlated with No-go > Go N2 difference scores in the whole sample of participants (Table 2; Figure 2). As expected, the size of the No-go > Go N2 difference was found to be associated to increased activation in a broad network of frontal and prefrontal cortices, predominantly in the left hemisphere.

**Table 2:** **Cortical areas identified to significantly correlate with No-go > Go N2 difference scores in the whole sample of participants**.

**Lobe**	**Structure**	***BA***	***K***	***X***	***Y***	***Z***	***T***
L Limbic lobe	Anterior cingulate	32	33	−5	15	35	−5.46
L Limbic lobe	Cingulate gyrus	24	23	−5	10	30	−6.17
L Frontal lobe	Superior frontal gyrus	11	20	−20	65	−10	−5.48
L Frontal lobe	Superior frontal gyrus	10	60	−20	65	0	−6.04
L Frontal lobe	Precentral gyrus	9	43	−35	5	40	−5.09
L Frontal lobe	Superior frontal gyrus	8	56	−20	25	50	−5.72
L Frontal lobe	Precentral gyrus	6	108	−35	0	30	−5.35
R Frontal lobe	Medial frontal gyrus	8	14	0	20	50	−4.70
R Limbic lobe	Cingulate gyrus	24	26	5	10	30	−7.79
R Limbic lobe	Cingulate gyrus	32	24	0	15	35	−5.69

BA, Brodmann area; K, cluster size; X, Y, Z, Spatial coordinates; T, contrast statistic.

All p's < 0.001.

Moreover, the correlation between the No-go > Go difference and peak voxel activation was significantly larger for the HC group than for the CDI group in the left BA10 area (*r* = 0.57 and –0.11, for HC and CDI, *p* < 0.01) and the left BA11 area (*r* = 0.61 and 0.11, for HC and CDI, *p* = 0.03). This suggests an abnormal functioning of these areas in the CDI group.

#### Now or later task

The number of trials in which the decision favored the immediate-reward option in the *now or later* task was taken as the main measure of sensitivity (intolerance) to reward delay. Mean (SD) scores were 10.52 (3.75), 17.00 (4.86), and 15.15 (4.59), for HC, PG, and CDI, respectively. The group effect was strongly significant [*F*_(2, 64)_ = 12.72, *MSE* = 19.38, *p* < 0.01]. Šidák-corrected *post-hoc* tests revealed significant differences between HC and PG (*p* < 0.01), and between HC and CDI (*p* < 0.01), but not between CDI and PG (*p* = 0.46).

Restricted linear regression analyses with severity as independent variable, and sensitivity to reward delay as dependent measure, were carried out for the PG and the CDI groups. In none of the two cases was severity significantly predictive of elevated sensitivity to reward delay [*t*_(20)_ = 1.06, *p* = 0.30; and *t*_(19)_ = 1.89, *p* = 0.08, for PG and CDI groups, respectively].

### Relationships between impulsivity dimensions and decision-making tasks

For the Go/No-go task, MANCOVAs across blocks, with the five UPPS-P dimensions as continuous covariates, were carried out on false alarm rates, hit rates, and hit latencies. No effects on false alarm and hit rates were close to significance. However, lack of premeditation and lack of perseverance exerted significant effects on latencies [Wilks' Λ = 0.74, *p* = 0.04, and Wilks' Λ = 0.60, *p* < 0.01, respectively]. The effect of lack of premeditation was restricted to block 4 [*F*_(1, 58)_ = 4.78, *MSE* = 610.59, *p* = 0.03], and the effect of lack of perseverance to block 2 [*F*_(1, 58)_ = 9.81, *MSE* = 830.74, *p* < 0.01]. The partial correlation between lack of premeditation and hit latency in block 4 (controlling for the other UPPS-P dimensions) was *r* = 0.38. Complementarily, the partial correlation between lack of perseverance and hit latency in block 2 was *r* = 0.28. In both cases, impulsivity significantly slowed decisions down.

Finally, the five UPPS-P dimensions were used as predictors of sensitivity to reward delay in a stepwise regression analysis. Negative urgency emerged as the only significantly predictive dimension [β = 0.43, *t*_(63)_ = 3.74, *p* < 0.01] included in the regression model.

## Discussion

Our general research aim was to test whether emotional and non-emotional dimensions of impulsivity were differentially predictive of decision-making and addictive behavior in three samples of PGs, CDIs, and HCs.

The first specific research target was to use impulsivity dimensions to *postdict* belongingness to clinical categories, and to check for correlations between impulsivity dimensions and gambling episodes/cocaine dosage exposure. With that aim in mind, regression analyses were carried out to estimate the value of impulsivity dimensions as postdictors of the clinical category (addicts vs. non-addicts, and PG vs. CDI). Only negative urgency (but not positive urgency), lack of premeditation (in a positive direction), and sensation seeking (in a negative direction) were indicative of selective inclusion in the groups of addicted individuals.

Although none of the dimensions discriminated between the two clinical groups (CDI, PG), an important difference between them emerged when the same dimensions were used as postdictors of addiction severity. Negative urgency independently covaried with gambling severity in PG, but did not predict cocaine use severity in CDI. This is consistent with previous reports that negative urgency is a sign of *overpathologization* in addictive processes (e.g., Michalczuk et al., 2011), but not with those in which negative urgency has been linked to cocaine neurotoxic effects. Although some recent works hypothesize a relationship between cocaine dependence severity and negative urgency (Albein-Urios et al., 2012a; Cándido et al., 2012), direct evidence of such a relationship is sparse (Verdejo-García et al., 2007a). Thus, the question of whether negative urgency is involved in overpathologization of different types of addictive processes, or has a privileged role in gambling remains open. Still, the neat relationship between gambling severity and negative urgency discards the possibility that such a relationship is exclusively mediated by cocaine neurotoxicity.

Our second research target was to check for the existence of differences between clinical and non-clinical groups, as well as exposure-mediated effects on performance in Go/No-go and delay-discounting tasks, and on evoked EEG activity during the Go/No-go task. The results from previous studies suggest that cocaine use, but not pathological gambling, relates to performance deficits in response inhibition skills (see Fillmore and Rush, 2002; Verdejo-García et al., 2007b; Kertzman et al., 2011; Van Holst et al., 2012). Accordingly, our results show that CDIs, but not PGs, perform abnormally in the Go/No-go task. CDIs presented focal increases of false alarms (commission errors). In parallel, and most importantly, ERP results showed an abnormal pattern of Go/No-go stimuli-evoked EEG activity in the CDI group (the No-go > Go N2 difference in the CDI group was the smallest among the three groups). The deleterious effect on N2 was mediated by cocaine dosage exposure. This pattern of results is compatible with the well-known association between cocaine consumption and malfunctioning of motor inhibition mechanisms (Kaufman et al., 2003; Garavan et al., 2008), and also with the proposal that such malfunctioning is at least partially due to neurotoxic dosage exposure effects.

In contrast to this difference between clinical groups found in the Go/No-go task, both CDIs and PGs were more sensitive than HCs to reward delay, as measured by the now-or-later decision-making task. In this case, neither the two groups of addicts differed between them, nor the effect was mediated by cocaine use or gambling severity. Consequently, the finding by Kertzman et al. (2011) that gamblers discount reward more rapidly than other addicts has not been replicated.

Results on source localization complemented the ones on the N2 ERP. The areas involved in the generation of the No-go > Go N2 difference (in the whole sample) are mostly coincident with the ones described in previous works. Reporting the involvement of pre-SMA and anterior cingulate is common to virtually all relevant studies. A variety of other dorsal, lateral, and anterior areas of the prefrontal cortex have been reported to functionally interact with these (Rubia et al., 2001; Horn et al., 2003; Nieuwenhuis et al., 2003; Lavric et al., 2004; Ridderinkhof et al., 2004a,b; Tanji and Hoshi, 2008; Zheng et al., 2008; Smith et al., 2013) in the generation of the No-go > Go N2 difference.

Also with regard to source location, some mention needs to be made about group differences. As noted above, left BA10 and BA11 were unique at correlating with the N2 differential effect in the control group, but not in the CDI group (which is suggestive of abnormal functioning of these areas in cocaine addicts). Among the different areas involved in Go/No-go performance, Horn et al. (2003) specifically attributed a temporally early, inhibitory role to BA10/11. Zheng et al. (2008), on the other hand, attributed a similar inhibitory role to left BA10. Importantly, none of the areas mentioned by Horn et al. as potentially involved in the emotional aspects of Go/No-go performance (posterior orbitofrontal cortex, the temporal poles, and the posterior cingulate) are involved either in differential N2 generation, or in HC-CDI source location differences in the present study. This pattern of data thus suggests that the deleterious effect of cocaine addiction on the Go/No-go task mainly affects its inhibition component.

The last research target was aimed at completing the triadic mapping between addictions, impulsivity, and decision-making. Therefore, we measured the impact of impulsivity on decision making, without taking addiction into account. Lack of premeditation and lack of perseverance slowed down responses in the Go/No-go task. Although such a result seems counterintuitive, previous results show that, in speeded decision-making tasks, impulsivity interferes with response selection. For example, Expósito and Andrés-Pueyo (1997) found that “more impulsive Ss are more affected by stimulus-response incompatibility and therefore present higher latencies” (p. 696). Further implications of such a result go beyond the aims of the present study^1^.

Still, the impulsivity dimensions involved in the latency effect (lack of premeditation and lack of perseverance), were only partially coincident with the ones related to addiction (lack of premeditation, sensation seeking, and negative urgency), and not coincident at all with the one predicting gambling severity (negative urgency). On the other hand, the impulsivity dimension predicting sensitivity to reward delay was the same one found to be predictive of gambling severity (negative urgency). Both the lack of involvement of emotional dimensions in Go/No-go performance, and the involvement of negative urgency in reward delay sensitivity are partially contradictory with Cyders and Coskunpinar's (2011a; although see Cyders and Coskunpinar, 2011b) findings on the relationship between trait and neuropsychological measures of impulsivity. Although our source location and behavioral results are fully congruent with each other, this apparent dissonance probably deserves further investigation.

In summary, among the emotional dimensions of impulsivity, negative urgency (but not positive urgency) has been observed to be selectively involved in addiction, independently of its type (pathological gambling, cocaine dependence). The tight link between negative urgency and emotionally-charged decision-making processes is reinforced by the fact that negative urgency was the only dimension significantly predicting sensitivity to reward delay in the delay discounting task. Furthermore, negative urgency was specifically related to gambling overpathologization. This effect is compatible with the possibility that gambling (large amounts of money for long periods) is fueled by negative emotions and moods, and such emotions and moods might operate as gambling triggers (Oakes et al., 2011, 2012; Williams et al., 2012). And, the other way round, it is also compatible with the possibility that dosage-like exposure to gambling episodes generates sensitization and neuroadaptive effects (Robinson and Berridge, 2003; Mathewson, 2009).

Results on sensation seeking probably deserve a short digression. Rather counterintuitively, sensation seeking scores were lower in both samples of addicted individuals, and were independently and inversely indicative of belongingness to any of the clinical samples. As noted in the introduction, sensation seeking has been shown to be linked to reward delay sensitivity, and to be a significant predictor of recreational, non-clinical drug use (Cyders and Coskunpinar, 2011a,b; Torres et al., submitted). This can be taken as independent evidence of the transition from positive to negative emotion-driven impulsivity in drug-use pathologization.

Among the non-emotional dimensions of impulsivity, lack of premeditation was found to be simultaneously involved in the two types of addiction, and in delaying Go/No-go decisions. However, in agreement with previous evidence, the effect of addiction on the Go/No-go task was limited to CDI, and was of a different nature (namely, an increased focal false alarm rate, and abnormal Go/No-go stimulus evoked cortical activity). In other words, the effects of impulsivity on Go/No-go decision-making and addictive processes involve different neurocognitive mechanisms. Impulsivity is not by itself responsible for the decision-making anomalies in cocaine addicts revealed by the Go/No-go task.

This study holds relevant strengths and noteworthy limitations. Some limitations are: first, the relatively small sample size of the clinical groups, which may have impacted the statistical power of multivariate contrasts. Secondly, the number of valid trials in the Go/No-go task (particularly, in the case of No-go trials) was too small to analyze ERPs dynamically, that is, trying to capture the changes in cortical activity occurring during the task (in a block-by-block fashion, or across phases). Unfortunately, any attempts to analyze the task in parts rendered the signal-to-noise ratio too low to capture any significant effect. And finally, it is important to mention the limitation of the current design to capture causality in the associations between emotional and non-emotional pathways to impulsive personality and impulsive action and choice and drug use behavior. As acknowledged in the introduction, such causal relationship can only be tracked by longitudinal studies. On the other hand, its major strengths are the use of a multidimensional approach to impulsive traits and impulsive decisions, measuring both emotional and non-emotional pathways to impulsive action and impulsive choice, and combining behavioral and electrophysiological approaches. Another relevant strength is the comparison between two clinical groups of addicted individuals who were matched in terms of baseline cognition (IQ) and clinical features, but strikingly differed in their impulsive action patterns, which, as discussed, have relevant implications both from the basic science and the clinical perspectives.

## Final remarks

The results presented here make two relevant contributions to the current literature. First, they show that aspects of impulsivity with a core component of negative emotion processing and appraisal play a key role in decision making (reward delay sensitivity), and addiction (gambling severity and intensity). And, secondly, they show that some key aspects of decision-making and neurobehavioral anomalies in addicts are independent of such factors. CDIs, but not PGs, performed abnormally in the Go/No-go task, and showed an abnormal N2 signal. Source location analyses show the involvement of a broad network of frontal and prefrontal areas (but not of prelimbic and limbic areas) in the generation of N2, as well as left BA10 and BA11 abnormal involvement in N2 in the group of cocaine addicts. This pattern of neurophysiologic results is in agreement with the existence of an association between cocaine consumption and malfunctioning of inhibition mechanisms, at least partially due to neurotoxic, cocaine exposure effects.

## Funding

The research presented here has been funded by grants from the Ministerio de Ciencia e Innovación, MICINN (Spain), for Ana Torres/José C. Perales (ref. # PSI2009-13133), and Andrés Catena/Antonio Maldonado (ref. # PSI2009-12217); by a Junta de Andalucía (Spain) grant (ref. # PB09-SEJ4752) for Antonio Cándido: and by a RETICS (Redes Temáticas de Investigación Cooperativa en Salud) subprogramme grant (Ref. # RD12/0028/0017) from the Ministerio de Sanidad, Servicios Sociales e Igualdad (Spain), for José C. Perales/Antonio Verdejo-García.

### Conflict of interest statement

The authors declare that the research was conducted in the absence of any commercial or financial relationships that could be construed as a potential conflict of interest.

## Footnotes

^1^In short, it seems to support the idea that impulsivity affects the decisional aspects of response selection in the Go/No-go task. This interpretation goes against the idea of motor impulsivity as mere disinhibition, namely, the malfunctioning of a central inhibitory mechanism (see also Donkers and Van Boxtel, 2004; Spinella, 2004; Perales et al., 2009, for discussions on the matter).
