# Corrigendum: Emotional and non-emotional pathways to impulsive behavior and addiction

**DOI:** 10.3389/fnhum.2014.00411

**Published:** 2014-08-22

**Authors:** José C. Perales

**Affiliations:** Learning, Emotion, and Decision Research Group, Mind, Brain and Behavior Research Center/Centro de Investigación Mente, Cerebro y Comportamiento, Universidad de GranadaGranada, Spain

**Keywords:** impulsivity, emotion, addiction, decision-making, delay discounting, Go/No-go, UPPS-P, N2 ERP

The responses of the control group to the now-or-later task were erroneously transcribed into the statistical software. Therefore, some results of the analyses involving this task are incorrect. The instances of the published manuscript affected by this error must be rewritten as follows.

## Now or later task

Mean (*SD*) scores were 16.48 (3.75), 17.00 (4.86), and 15.15 (4.59), for HC, PG, and CDI, respectively. The group effect was not significant [*F*_(2, 64)_ < 1] (in contrast to what was reported in the published version of the paper, p. 7, l. 1).

## Relationships between impulsivity dimensions and decision-making tasks

The five UPPS-P dimensions were used as predictors of sensitivity to reward delay in a stepwise regression analysis. Sensation seeking (instead of negative urgency, as stated in the published version, p. 7, l. 30) emerged as the only significantly predictive dimension [β = −0.30, *t*_(63)_ = −2.48, *p* < 0.02].

## Discussion

This error only affects secondary analyses, and therefore its impact on general conclusions is rather limited. Still, we argued that “the involvement of negative urgency in reward delay sensitivity are partially contradictory with Cyders and Coskunpinar's (2011a; although see Cyders and Coskunpinar, 2011b) findings on the relationship between trait and neuropsychological measures of impulsivity” (p. 8). Given that negative urgency is not really involved in reward delay sensitivity, this contradiction no longer exists. Similarly, we mentioned that “the tight link between negative urgency and emotionally-charged decision-making processes is reinforced by the fact that negative urgency was the only dimension significantly predicting sensitivity to reward delay in the delay discounting task.” (pp. 8–9). This assertion is not supported by the corrected delay discounting data. Actually, sensation seeking was the only impulsivity dimension independently (and inversely) predicting reward delay sensitivity.

### Conflict of interest statement

The author declares that the research was conducted in the absence of any commercial or financial relationships that could be construed as a potential conflict of interest.
